# Optimizing proximal fibula tumor resection: “The open door technique” for better surgical access and outcomes - A case series^☆^

**DOI:** 10.1016/j.ijscr.2025.111992

**Published:** 2025-09-27

**Authors:** Sadegh Saberi, Hamed Naghizadeh, Sepand Heydari, Elham Poorghasem, Mehdi Bayati, Seyyed Saeed Khabiri

**Affiliations:** a Joint Reconstruction Research Center, Department of Orthopedics, Tehran University of Medical Sciences, Iran; b Department of Pediatrics, Children's Medical Center, Tehran University of Medical Sciences, Tehran, Iran

**Keywords:** Proximal fibula, Limb-sparing surgery, Orthopedic oncology, Surgical technique, Surgical outcomes

## Abstract

**Objective:**

Proximal fibula tumors pose significant surgical challenges due to their proximity to vital neurovascular structures, including the common peroneal nerve. Traditional resection techniques often require extensive tissue manipulation, which can lead to complications such as peroneal nerve injury and knee instability. This study introduces and evaluates the “open door technique,” designed to enhance surgical visualization and access, thereby reducing complications while achieving oncologic control.

**Methods:**

We conducted a retrospective cohort study of 31 patients who underwent proximal fibula tumor resection using the “open door technique” from 2015 to 2022. Patient demographics, tumor characteristics, and functional outcomes were documented. Preoperative imaging and a standardized surgical approach were employed, with follow-up assessments including the Musculoskeletal Tumor Society (MSTS) score and the Toronto Extremity Salvage Score (TESS). Descriptive statistics and comparative analyses were applied to evaluate surgical and functional outcomes.

**Results:**

The “open door technique” achieved negative resection margins in 93.5 % of cases, with a recurrence rate of 6.5 % over a mean follow-up period of 36 months. Postoperative complications included permanent peroneal nerve injury in 12.9 % of patients and transient nerve injury in 9.7 %, both lower than rates typically associated with traditional techniques. Functional outcomes were favorable, with 87.1 % of patients achieving good to excellent MSTS and TESS scores, indicating practical neurovascular and ligamentous structure preservation.

**Conclusion:**

The “open door technique” provides an effective, function-sparing approach for proximal fibula tumor resection, balancing oncologic control with reduced neurovascular complications and improved functional outcomes. These findings support its potential as a valuable addition to orthopedic oncology practices. Further studies are recommended to confirm its applicability across a broader patient population.

## Highlights

• The “open door technique” significantly improves surgical visualization, allowing safer dissection and better preservation of neurovascular structures.
• Negative resection margins were achieved in 93.5% of cases, indicating strong oncologic control.
• Peroneal nerve complications were reduced compared to traditional methods, with only 12.9% experiencing permanent injury.
• Functional outcomes were favorable, with over 87% of patients achieving good to excellent MSTS and TESS scores.
• Local recurrence was low (6.5%), and no distant metastases were observed during a mean follow-up of 36 months.

## 1 Introduction

Proximal fibula tumors, though relatively rare, present unique surgical challenges due to the complex anatomy of the region. These tumors account for roughly 2.4 % of all lower extremity bone sarcomas, with osteochondroma and chondrosarcoma among the most frequently encountered types [1,2]. Surgical management is complicated by the proximity of these tumors to critical neurovascular structures, including the common peroneal nerve and the anterior and posterior tibial vessels [3]. Traditional resection techniques aim to achieve negative margins, yet the necessity of preserving neurovascular integrity in this area leads to frequent complications [4]. Studies have reported rates of peroneal nerve injury as high as 13.5 % [5], with some earlier data indicating up to 33 % in complex cases [6]. Such complications often result in significant functional impairments, like foot drop, impacting patients' quality of life [7].

Various surgical approaches to proximal fibula tumors have evolved, balancing oncologic safety with functional preservation. Malawer's classification, which guides surgical resection types, emphasizes preserving knee stability and neurovascular structures [8]. However, traditional techniques frequently fall short, often necessitating extensive soft tissue manipulation that can compromise outcomes. Limited visibility of posterior structures during resection has further contributed to suboptimal results, including recurrence and accidental neurovascular damage [9]. These limitations underscore the need for improved surgical approaches to visualize critical anatomy better while minimizing injury to surrounding tissues.

**Fig. 1:**
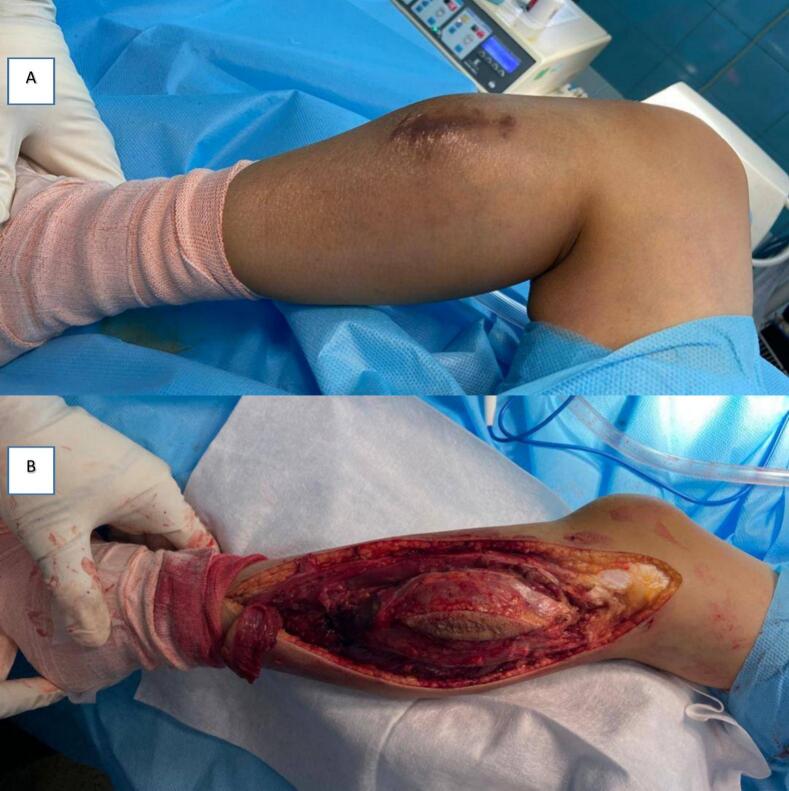
A: Patients were positioned supine under general anesthesia with lateral support provided for stability. B: Optimal exposure and precise separation of the tumor mass.

In response to these challenges, we introduce the “open door technique,” a novel approach designed to enhance visualization and improve access to proximal fibula tumors. Creating a hinged segment of the fibula offers surgeons controlled access to the tumor, enabling meticulous dissection and preservation of neurovascular structures. This study aims to evaluate the oncologic and functional outcomes of the “open door technique,” comparing its safety and efficacy to conventional resection methods. Through this analysis, we hope to establish a more effective protocol for managing proximal fibula tumors that prioritizes oncologic control and functional preservation (Fig. 1).

## 2 Materials and methods

This study is a retrospective cohort analysis conducted at a single institution to evaluate the outcomes of the “open door technique” for proximal fibula tumor resection. Ethical approval was obtained from the Institutional Review Board (IRB) before study initiation, and informed consent was secured from all patients for their participation and the use of their medical data. All procedures adhered to the principles of the Declaration of Helsinki, ensuring patient confidentiality and data protection.

From 2015 to 2022, we identified 31 patients who met the inclusion criteria, which encompassed adults aged 16 to 65 years, with a mean age of 33, diagnosed with either malignant tumors (such as osteosarcoma and Ewing's sarcoma) or aggressive benign lesions (such as giant cell tumors). Patients with prior surgeries in the proximal fibula, non-tumor-related pathologies, or insufficient imaging records were excluded to maintain consistency in preoperative assessment and surgical approach. Each patient underwent detailed preoperative imaging, including magnetic resonance imaging (MRI) and computed tomography (CT) scans, to evaluate the tumor's extent and proximity to critical neurovascular structures. These imaging assessments were reviewed by a multidisciplinary team, including radiologists and orthopedic oncologists, to minimize observer bias and ensure precise surgical planning.

The “open door technique” was performed with patients under general anesthesia in a supine position, with lateral support for stability. A longitudinal incision extended from the lateral femoral condyle to the mid-shaft of the fibula, providing broad exposure to the surgical field. The common peroneal nerve was carefully identified below and posterior to the biceps femoris tendon, then dissected free from surrounding tissue to prevent injury. After identifying the nerve, the peroneal muscles were detached proximally at the tibiofibular joint, and a distal osteotomy was executed just beyond the tumor's location. The fibular segment was rotated outward using a bone holder, creating an “open door” effect that improved access to the anterior tibial vascular bundle and other neurovascular structures essential for the procedure. Tumor resection was conducted en bloc, with careful inward manipulation and elevation of the bone and mass, allowing for precise preservation of the neurovascular pathways of the tibialis posterior. In cases where lateral ligament complex resection was necessary, the biceps femoris tendon and lateral collateral ligament were reattached to the proximal tibia to maintain knee stability. Hemostasis was achieved through bipolar cautery and vessel ligation, and postoperative knee immobilization in 30 degrees of flexion was applied for two weeks, followed by a structured physical therapy program to promote recovery.

Postoperative follow-up included monitoring for complications, such as peroneal nerve palsy, wound healing problems, knee instability, and tumor recurrence. Patients underwent regular imaging (MRI or CT scans) at six-month intervals during the first two years and then annually, with a mean follow-up duration of 36 months (24–60 months). Functional outcomes were assessed using the Musculoskeletal Tumor Society (MSTS) score and the Toronto Extremity Salvage Score (TESS) at three, six, and twelve months post-surgery to monitor recovery progression. An orthopedic specialist, blinded to the surgical procedures, conducted these evaluations to reduce assessment bias.

Descriptive statistics were used to analyze demographic and clinical variables, presented as frequencies, percentages, means, and standard deviations where appropriate. Functional outcome scores (MSTS and TESS) are reported as mean values with standard deviations. For comparative purposes, the “open door technique” outcomes were assessed against historical data from conventional resection techniques in the literature. Categorical outcomes were analyzed using the chi-square test, while continuous variables were compared using *t*-tests, with statistical significance set at *p* < 0.05. This case series has been reported in line with the PROCESS 2025 guideline [10].

## 3 Results

This study evaluated the surgical and functional outcomes of 31 patients who underwent proximal fibula resection using the “open door technique” between 2015 and 2022. Patient demographics, tumor characteristics, and surgical details are summarized to provide a clear context for the findings.

### 3.1 Patient demographics and tumor characteristics

The study cohort comprised 31 patients ranging in age from 16 to 65 years, with a mean age of 33.3 years (SD ± 6.3). Eighteen patients were male (58.1 %) and thirteen were female (41.9 %). Tumor histology included osteogenic sarcoma in 12 patients (38.7 %), Ewing's sarcoma in 7 patients (22.6 %), giant cell tumor (GCT) in 8 patients (25.8 %), and osteochondroma in 4 patients (12.9 %) (Table 1).

**Table 1:** Demographic and tumor-related characteristics of the study population.

Characteristic	Number of patients	Percentage (%)
Total patients	31	100
Age		
16–25 years	10	32.3
26–35 years	8	25.8
36–45 years	7	22.6
46–55 years	4	12.9
56–65 years	2	6.4
Mean age	33.32 ± 6.3 years	
Gender		
Male	18	58.1
Female	13	41.9
Tumor types		
Osteogenic sarcoma	12	38.7
Ewing's sarcoma	7	22.6
Giant cell tumor (GCT)	8	25.8
Osteochondroma	4	12.9

### 3.2 Surgical outcome

The “open door technique” was successfully executed in all cases. The average operative time was 112 min (range: 70–150 min), and the mean intraoperative blood loss was 120 mL (range: 80–300 mL). Notably, resection margins were classified as negative in 29 patients (93.5 %), ensuring a high rate of oncologic control, with two cases (6.5 %) reporting close margins (<1 mm) without evidence of tumor invasion. No intraoperative injuries to the anterior or posterior tibialis vessels were documented, highlighting the technique's efficacy in preserving critical neurovascular structures (Table 2).

**Table 2:** Surgical and functional outcomes assessed by MSTS and TESS scores.

Outcome measures	Number of patients	Percentage (%)
Complications		
Peroneal nerve sacrifice	4	12.9
Transient peroneal nerve damage	3	9.7
Relapse	2	6.45
Wound healing issues	3	9.7
Knee instability	2	6.45

Functional outcomes (MSTS Score)		
Excellent (90–100 %)	21	67.7
Good (80–89 %)	6	19.4
Fair (70–79 %)	3	9.7
Poor (<70 %)	1	3.2

Functional outcomes (TESS Score)		
Excellent (90–100 %)	19	61.3
Good (80–89 %)	8	25.8
Fair (70–79 %)	3	9.7
Poor (<70 %)	1	3.2

### 3.3 Complications

Complications observed postoperatively included peroneal nerve involvement in seven patients (22.6 %): in four cases (12.9 %), the peroneal nerve required surgical sacrifice due to extensive tumor involvement, resulting in permanent foot drop. These patients adapted with the use of ankle-foot orthoses (AFOs). Additionally, transient peroneal nerve damage was observed in three patients (9.7 %), all of whom achieved full recovery within five months.

**Fig. 2:**
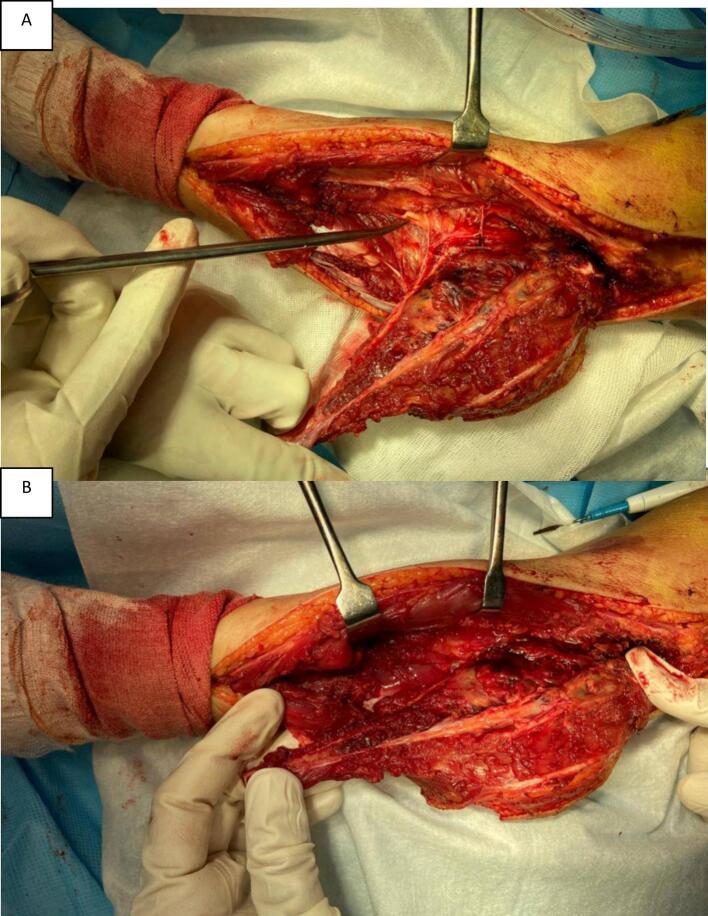
A: External rotation of the tumor and fibula creates an “open door” providing clear exposure of the neurovascular bundle. B: Meticulously dissect and preserve the neurovascular bundle.

Wound healing complications were reported in three patients (9.7 %), including minor infections and delayed wound closure. These issues were effectively managed with conservative treatments, such as antibiotics and extended wound care. Knee instability, a concern in lateral ligament complex resection cases, developed in two patients (6.5 %). One case was successfully managed with ligament reconstruction, while the other required long-term knee brace use for stability (Fig. 2).

### 3.4 Oncologic outcomes

At a mean follow-up of 36 months (range: 24–60 months), the study documented a local recurrence rate of 6.5 % (2 out of 31 patients), specifically in patients with high-grade osteosarcoma. Both recurrence cases were subsequently managed with additional wide resection. Importantly, no distant metastases were detected during the follow-up period, resulting in a three-year disease-free survival rate of 93.5 %.

### 3.5 Functional outcomes

**Fig. 3:**
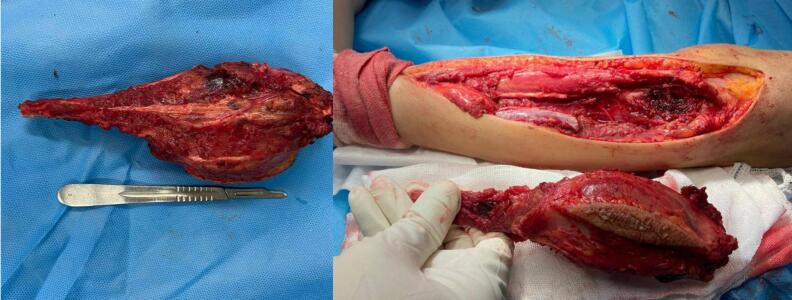
A: The tumor was completely resected. B: Ensuring the neurovascular structures remained intact.

Functional recovery was assessed using the MSTS and TESS scores at multiple follow-up intervals. MSTS scores indicated excellent functional outcomes (90–100 %) in 21 patients (67.7 %), good outcomes (80–89 %) in six patients (19.4 %), fair outcomes (70–79 %) in three patients (9.7 %), and a poor outcome (<70 %) in one patient (3.2 %). TESS scores corroborated these findings, with 19 patients (61.3 %) achieving excellent scores, eight patients (25.8 %) with good scores, three patients (9.7 %) with fair scores, and one patient (3.2 %) with poor scores. These results suggest that the “open door technique” enables high rates of functional preservation and recovery in the majority of patients (Fig. 3).

## 4 Discussion

The “open door technique” for proximal fibula tumor resection demonstrates a promising balance between achieving oncologic control and preserving function, addressing the challenges of operating within the complex anatomical region of the proximal fibula. This technique enhances the surgeon's ability to visualize and protect critical neurovascular structures, reducing the risk of complications traditionally associated with proximal fibula resections. Our findings suggest that this approach provides an effective alternative to traditional methods, yielding favorable functional outcomes and a low complication rate.

The open door technique is particularly indicated in cases where proximal fibula tumors are in proximity to critical neurovascular structures, such as the common peroneal nerve or anterior tibial vessels, and enhanced visualization is required to achieve safe margins. It is also suitable for patients who prioritize preserving knee stability and neurovascular integrity, such as younger and more active individuals. The technique can also be considered for malignant and aggressive benign tumors.

Conversely, the open door technique may be less suitable in cases where the tumor extends into the proximal tibia, preventing adequate mobilization of the fibular segment required for the hinged approach. It is also contraindicated when tumor extension involves major vascular structures that cannot be safely dissected with this method, or when local soft tissue conditions hinder the controlled outward movement of the fibula. In such circumstances, more radical resection techniques may be warranted.

Traditional resection techniques for proximal fibula tumors, particularly Malawer resections, frequently sacrifice important structures such as the peroneal nerve and lateral ligament complex to achieve adequate margins. Malawer Type I and II resections, which involve the excision of the fibular head and surrounding tissue, often result in complications, including foot drop, knee instability, and significant functional impairment. Studies have documented that these complications occur at rates as high as 22 % for peroneal nerve palsy and 12–25 % for knee instability, reflecting the challenges of safely managing neurovascular structures in this region [8,11].

In contrast, the “open door technique” offers a nuanced approach, hinging the fibula outward to allow targeted access to tumor margins without extensive resection of surrounding soft tissues. Our results show that permanent peroneal nerve injury occurred in only 12.9 % of cases, with an additional 9.7 % experiencing transient nerve injury. These rates mark a substantial improvement over those associated with traditional Malawer techniques, as Erler et al. [6] reported. This enhanced visualization afforded by the “open door” maneuver supports meticulous dissection, likely contributing to this study's reduced incidence of neurovascular injury.

Reserving knee stability is critical in surgeries involving the proximal fibula, as disruption of the lateral ligament complex and biceps femoris tendon attachment can lead to postoperative instability. Traditional techniques that necessitate resection of these structures often compromise stability, requiring subsequent ligament reconstruction or brace support [8]. Our results indicate that the “open door technique” allows for selective preservation or reattachment of these ligaments, reducing the likelihood of knee instability. In this study, only 6.5 % of patients experienced postoperative knee instability, underscoring the technique's advantage in maintaining functional integrity.

Functional assessment using MSTS and TESS scores showed that most patients achieved excellent to good outcomes, with mean MSTS scores above 80 %, suggesting a high level of functional preservation. This aligns with findings from studies such as Inatani et al., which demonstrate that preserving the peroneal nerve and stabilizing structures significantly improves postoperative function [12,13]. Thus, the “open door technique” represents a practical approach to balancing oncologic safety with functional integrity, a priority in modern orthopedic oncology.

Achieving adequate oncologic margins is paramount in tumor resection surgeries to reduce the risk of recurrence [14]. In our study, negative resection margins were achieved in 93.5 % of cases, with a recurrence rate of only 6.5 % at a mean follow-up of 36 months. These findings compare favorably with recurrence rates associated with other conservative methods, such as marginal excision and curettage, which often yield higher recurrence rates, particularly in aggressive benign tumors like giant cell tumors (GCTs) [[15], [16], [17]]. By improving surgical visibility, the “open door technique” allows for precise resection with controlled margins, potentially lowering the risk of recurrence without necessitating radical resection.

Several limitations of this study warrant consideration. The retrospective design limits our ability to establish causative conclusions, and the relatively small, single-center sample restricts the generalizability of these findings. Additionally, while the three-year follow-up provides initial insights into recurrence and functional outcomes, longer-term studies are needed to capture the entire durability of the technique's benefits. Future research should focus on prospective, multi-center studies that directly compare the “open door technique” to established methods, such as Malawer resections and marginal excisions, regarding functional outcomes and recurrence rates. Further refinement of patient selection criteria is also crucial, as this technique may best suit specific tumor types or grades. Identifying these factors could optimize the “open door technique” for broader clinical use.

## 5 Conclusion

The “open door technique” introduces an innovative, function-sparing approach to proximal fibula tumor resection, emphasizing oncologic control and functional preservation. By enhancing surgical visualization and allowing for precise neurovascular dissection, this technique can reduce the risk of complications typically associated with proximal fibula resections. Our findings support the potential of the “open door technique” to become a valuable addition to orthopedic oncology protocols, offering patients improved quality of life post-surgery.

The patients provided informed written consent for print and electronic publication of this case series.

## Author contribution

**Sadegh Saberi** M.D.: Conceived and designed the study, led the research team and contributed to manuscript revision.

**Seyyed Saeed Khabiri** M.D.: Assisted in study design, collected the primary data, and contributed to data analysis.

**Sepand Heydari** M.D.: Provided critical revisions that were important for the intellectual content.

**Elham Poorghasem**: Literature review, Validation, Writing – review & editing.

**Mehdi Bayati** M.D.: Assisted in data collection and analysis.

**Hamed Naghizadeh** M.D.: Coordinated the research efforts, ensured the integrity of the work, and contributed to manuscript preparation and submission.

## Consent

Written informed consent was obtained from the patient for publication and any accompanying images. A copy of the written consent is available for review by the Editor-in-Chief of this journal on request.

## Ethical approval

The research was evaluated by the Research Ethics Committee of our University of Medical Sciences (IRB approval ID: IR.TUMS.IKHC.REC.1401.200). Patients signed informed consent before enrolment at the latest follow-up.

## Guarantor

Seyyed Saeed Khabiri M.D.

## Research registration number

This study does not qualify as a “First in Man” study and therefore did not require registration

## Funding

This research did not receive any specific grant from funding agencies in the public, commercial, or not-for-profit sectors. No sponsor had any role in the design, execution, interpretation, or writing of the study.

## Conflict of interest statement

All authors declare that there are no financial or personal relationships with other people or organizations that could inappropriately influence their work.
